# COVID-19 versus H1N1: challenges in radiological diagnosis—comparative study on 130 patients using chest HRCT

**DOI:** 10.1186/s43055-021-00455-8

**Published:** 2021-03-17

**Authors:** Ahmed Samir, Nagy N. N. Naguib, Abdelaziz Elnekeidy, Ayman Ibrahim Baess, Amal Shawky

**Affiliations:** 1 Department of Radio-diagnosis and Intervention, Faculty of Medicine, University of Alexandria, Alexandria, Egypt; 2 Institute for Diagnostic and Interventional Radiology, Johann Wolfgang Goethe University Hospital, Frankfurt am Main, Germany; 3 Department of Chest diseases, Faculty of Medicine, University of Alexandria, Alexandria, Egypt

**Keywords:** COVID-19, H1N1, HRCT

## Abstract

**Background:**

During the current second wave of COVID-19, the radiologists are expected to face great challenges in differentiation between COVID-19 and other virulent influenza viruses, mainly H1N1. Accordingly, this study was performed in order to find any differentiating CT criteria that would help during the expected clinical overlap during the current Influenza season.

**Results:**

This study was retrospectively conducted during the period from June till November 2020, on acute symptomatic 130 patients with no history of previous pulmonary diseases; 65 patients had positive PCR for COVID-19 including 50 mild patients and 15 critical or severe patients; meanwhile, the other 65 patients had positive PCR for H1N1 including 50 mild patients and 15 critical or severe patients. They included 74 males and 56 females (56.9%:43.1%). Their age ranged 14–90 years (mean age 38.9 ± 20.3 SD). HRCT findings were analyzed by four expert consultant radiologists in consensus. All patients with COVID-19 showed parenchymal or alveolar HRCT findings; only one of them had associated airway involvement. Among the 65 patients with H1N1; 56 patients (86.2%) had parenchymal or alveolar HRCT findings while six patients (9.2%) presented only by HRCT signs of airway involvement and three patients (4.6%) had mixed parenchymal and airway involvement*.* Regarding HRCT findings of airway involvement (namely tree in bud nodules, air trapping, bronchial wall thickening, traction bronchiectasis, and mucous plugging), all showed significant *p* value (ranging from 0.008 to 0.04). On the other hand, HRCT findings of parenchymal or alveolar involvement (mainly ground glass opacities) showed no significant relation.

**Conclusion:**

HRCT can help in differentiation between non-severe COVID-19 and H1N1 based on signs of airway involvement.

## Background

Human infection by coronavirus family was believed to be attributed to six major types, two types of which caused severe respiratory symptoms. The first is severe acute respiratory syndrome (SARS), seen in south China in 2003, and the other is Middle East respiratory syndrome (MERS), seen in Saudi Arabia in 2012 [1]. But in December 2019, novel coronavirus disease (COVID-19) was first described in Wuhan, China, then rapidly spread all over the world and announced as pandemic in March 2020 [2]. Another described human infection was attributed to influenza A (“swine” influenza or H1N1) virus that eventually result in some seasonal epidemics and unavoidable pandemics, last one in June 2009 [3].

Clinical picture of COVID-19 and H1N1 may overlap as both cause variable degrees of respiratory symptoms ranging from mild (flu like) up to severe (acute respiratory distress syndrome, ARDS) [3, 4]. Also blood picture in their laboratory analysis may overlap, as both tend to be associated with non-elevated white blood cell count (WBC) and low lymphocytic count [5, 6].

During the current second wave of COVID-19, the radiologists are expected to face great challenges in differentiation between COVID-19 and other virulent influenza viruses, mainly H1N1. Accordingly, this study was performed in order to find any differentiating CT criteria that would help during the expected clinical overlap during the current Influenza season.

## Methods

### Study population and medical records review

This study was retrospectively conducted, during the period from June till November 2020, on acute symptomatic 130 patients. Among them, 65 patients had positive PCR results for COVID-19 (registered during the period from February till May 2020), including 50 mild or moderate patients (with O_2_ saturation at room air > 93%) and 15 critical or severe patients (with O_2_ saturation at room air < 93% and needed external respiratory support). The other 65 patients had positive PCR results for H1N1 patients (registered during the period from October 2018 till November 2020), including 50 mild or moderate patients and 15 critical or severe patients as well. They included 74 males and 56 females (56.9%:43.1%). Their age ranged 14–90 years (mean age 38.9 years ± 20.3 SD).

The study was approved by The Ethics Committee of our University hospital. Patient consent was waived by the Research Ethics Board, assuring respect of confidentiality of the patients and medical records.

*Inclusion criteria were as follows*: (1) acute illness with respiratory symptoms either mild or severe based on the degree of hypoxia and the need for external respiratory support, (2) HRCT for positive COVID-19 patients, and (3) HRCT for positive H1N1 patients. All patients should have a confirmed PCR diagnosis of the disease entity in order to be enrolled in the study.

*Exclusion criteria were as follows:* (1) degraded quality of CT scans due to patient unavoidable tachypnea with respiratory motion artifacts; (2) past history of chronic airway disease such asthma, COPD, or bronchiectasis due to possible overlap with the CT findings of infection and expected bias; and (3) known patients with secondary bacterial infection, proved laboratory by culture/sensitivity tests.

### CT scanning and parameters

CT examinations were conducted using multiple MDCT machines including: GE LightSpeed Plus 4 slice CT scanner (USA), Philips Brilliant-16 (USA), Siemens SOMATOM Emotion 16 and Siemens SOMATOM Sensation 64 (Germany), Canon Medical Systems; Toshiba Aquilion 64 and Toshiba Aquilion CXL/CX 128 (USA).

CT scanning parameters were as follows: slice thickness, 1–2.5 mm; FOV = 350mm × 350 mm; tube rotation, 0.6–0.9 s; detector collimation, 1 mm; helical mode (volumetric HRCT); kVp and mA per slice, 120–130 kVp and 200–400 mA, according to the type of MSCT machine used; the weight of the patient; and the clinical indication.

Intra-venous contrast administration was done to 11 patients only as clinically requested. It was non-ionic iodinated contrast (350mg/ml concentration and 70–90 ml volume at 4–5 ml/s injection rate). No major reactions were reported. Only minor reactions were reported such as sense of hotness and nausea, which needed noting but assurance.

### CT analysis

CT images were assessed in consensus by four consultant radiologists (having long time experience in chest imaging; 10, 15, and 25 years). They were blinded to the final clinical diagnosis. Image analysis in axial, sagittal, and coronal planes was done using both maximum intensity projection (MIP) and minimum intensity projection (Min-IP) reconstructions. The following CT features were compared between each pathological process:

A) Site of the pathology: unilateral or bilateral—focal, multi-focal, or diffuse.
B) HRCT findings of pulmonary parenchymal or alveolar involvement including ground glass opacities (GGOs), consolidative changes, interlobular septal thickening, and “crazy paving pattern”.
C) HRCT findings of airway involvement including bronchial wall thickening, mucous plugging, and traction bronchiectasis/bronchiolectasis. This is in addition to secondary air trapping with or without “head cheese pattern.”
D) Relevant CT findings including pleural, pericardial, and nodal lesions.

### Statistical analysis

The prevalence rate of HRCT findings was estimated as the percentage of patients showing each criteria or abnormality. Data were compared using a chi-square test and *p* value < 0.05 was considered statistically significant. Sensitivity, specificity, positive predictive value (PPV), and negative predictive value (NPV) of HRCT were estimated for differentiation of H1N1 from COVID19.

## Results

Sixty-four patients with COVID-19 showed pure lung parenchymal or alveolar pattern of the disease. Only one patient (1.5%) presented by mixed parenchymal pattern and airway involvement (manifested by tree in bud nodules). Fifty-six patients with H1N1 (86.2%) showed parenchymal or alveolar pattern of the disease while six patients (9.2%) presented mainly with airway disease manifestations and three patients (4.6%) showed mixed parenchymal and airway involvement (Table 1).

**Table 1:** Distribution of COVID-19 and H1N1 patients according to predominant pathological/radiological pattern

Predominant pathological/radiological pattern:	COVID-19		H1N1	
	No	%	No	%
**[A] Parenchymal or alveolar involvement only:**	64	98.5%	56	86.2%
**Mild**	49 (76.6%)	75.4%	41 (73.2%)	63.1%
**Severe**	15 (23.4%)	(23.1%)	15 (26.8%)	(23.1%)
**[B] Airway involvement only:**	Not detected		6	9.2%
**Mild**	-	-	6 (100%)	9.2%
**Severe**	-	-	0	0%
**[C] Mixed parenchymal and airway involvement:**	1	1.5%	3	4.6%
**Mild**	1 (100%)	1.5%	3 (100%)	4.6%
**Severe**	-	-	-	-
**Total**	**65**	**100%**	**65**	**100%**

**Fig. 1:**
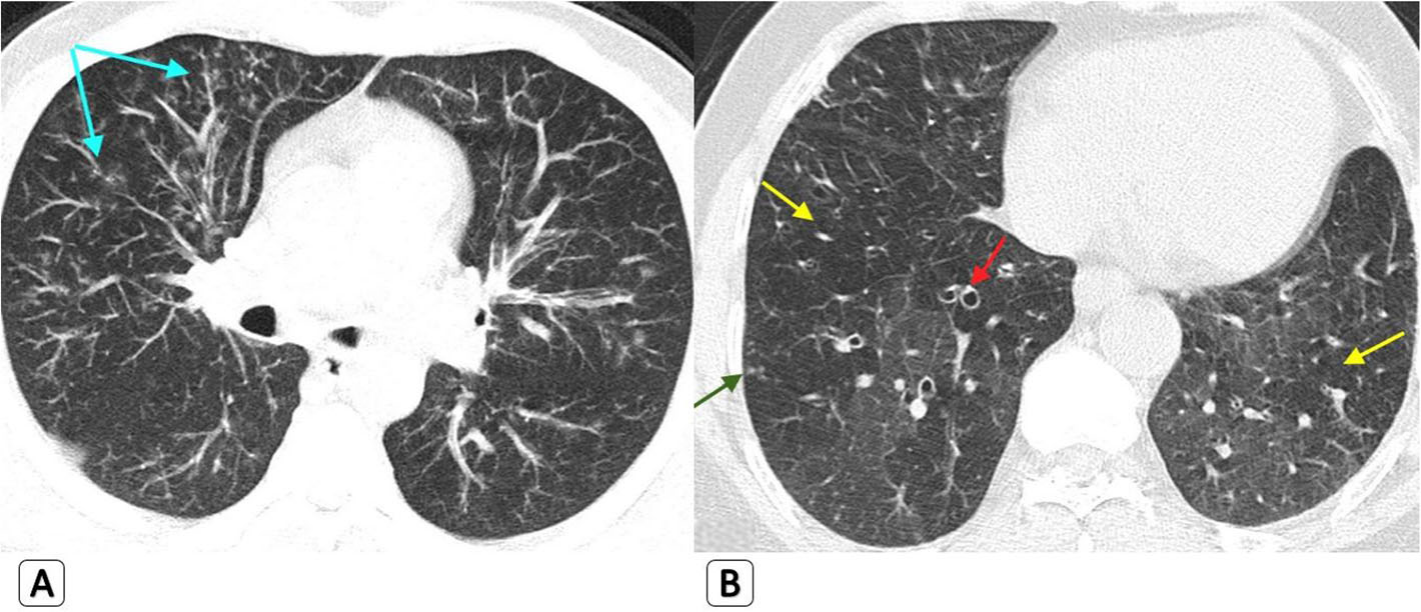
Mixed parenchymal/alveolar and airway abnormalities seen with H1N1: 47-year-old male H1N1 patient. **a** Axial HRCT chest lung window with “MIP” reconstruction demonstrating tree in bud nodules (blue arrows) and small ground-glass opacities. **b** Axial HRCT lung window showing mild bronchiectatic changes with “signet ring sign” (red arrows), patchy areas of mild air trapping (yellow arrows), and tiny few right lower sub-pleural tree in bud nodules (green arrow)

**Fig. 2:**
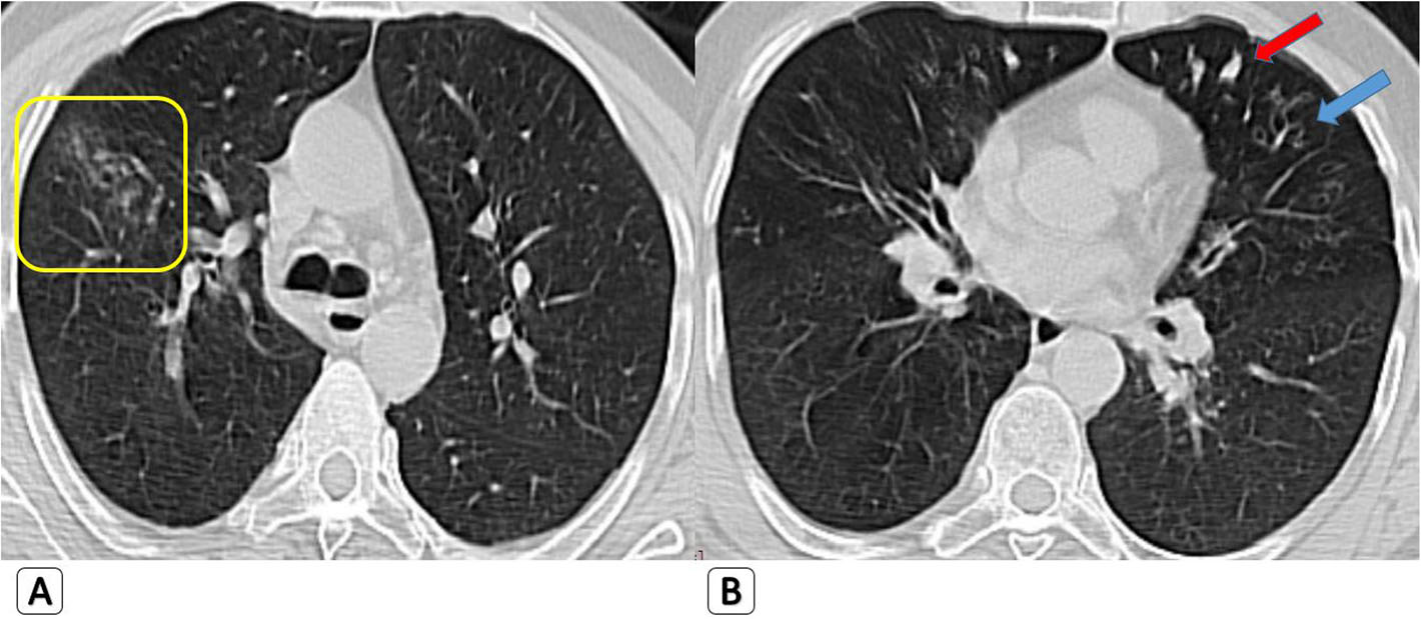
Mixed parenchymal/alveolar and airway abnormalities seen with H1N1: 37-year-old male H1N1 patient. **a** Axial HRCT chest lung window showing right sided mild alveolar ground glass nodular opacities (yellow square). **b** Axial HRCT lung window showing left lingular zone and to lesser extent right middle lobar air trapping with bronchiectatic changes (blue arrow) and mucous plugging (red arrow)

*Regarding the HRCT findings that were depicted only in H1N1 patients while absent in COVID-19 patients:* They are all related to airway involvement with mild clinical presentation. Bronchial wall thickening was seen in five patients with H1N1 (7.7%). Traction bronchiectasis was seen in five patients with H1N1 (7.7%). Mucous plugging was seen in four patients with H1N1 (6.2%). Air trapping was seen in seven patients with H1N1 (10.8%). “Head cheese pattern” with mixed air trapping and ground glass attenuation was seen in three patients with H1N1 (4.6%) (Figs. 1 and 2) (Table 2).

**Table 2:** Distribution of COVID-19 and H1N1 patients according to predominant HRCT finding with statistical analysis of significance

HRCT findings	COVID-19		H1N1		***X***^***2***^	***P*** value
	***N*** (65)	%	***N*** (65)	%		
**Site of the pathology:**						
**Bilateral**	55	84.6%	60	92.3%	1.88	0.17
**Unilateral**	10	15.4%	5	7.7%	1.88	0.17
**Mixed**	50	76.9%	55	84.6%	1.24	0.27
**Lower lobar only**	10	15.4%	7	10.8%	0.61	0.44
**Upper lobar only**	5	7.7%	3	4.6%	0.53	0.47
**Diffuse/mixed**	30	46.2%	35	53.8%	0.77	0.38
**Sub-pleural**	34	52.3%	30	46.2%	0.49	0.48
**Proximal**	1	1.5%	Not detected		1	0.32
**HRCT findings related to parenchymal or alveolar pathology (GGOs):**						
**Mild stage:**	**50**	**76.9%**	**44**	**67.7%**		
Pure ground glass nodules or patchy opacities (GGOs).	18 (36%)	27.7%	10 (22.7%)	15.4%	1.97	0.16
GGOs showing peripheral organization “Atoll sign”	13 (26%)	20%	7 (15.9%)	10.8%	1.42	0.23
GGOs mixed with consolidative changes.	19 (38%)	29.2%	27 (61.3%)	41.5%	5.11	0.02
**Severe or critical stage:**	**15**	**23.1%**	**15**	**23.1%**		
GGOs mixed with consolidative changes.	1 (7%)	1.5%	Not detected		1	0.31
GGOs mixed with fibrotic changes.	1 (7%)	1.5%	Not detected		1	0.31
“Crazy paving pattern” (part of DAD or ARDS)	13 (86%)	20%	15 (100%)	23.1%	2.14	0.14
**HRCT findings related to bronchial/bronchiolar airway pathology: (N.B: either isolated or mixed with alveolar pattern, all were mild)**						
Tree in bud nodules	1	1.5 %	9	13.8%	6.93	0.008*
Air trapping	Not detected		7	10.8%	7.4	0.007*
Bronchial wall thickening	Not detected		5	7.7%	5.2	0.02*
Traction bronchiectasis	Not detected		5	7.7%	5.2	0.02*
Mucous plugging	Not detected		4	6.2 %	4.13	0.04*
Mixed air trapping and GGO “Head cheese pattern”	Not detected		3	4.6%	3.07	0.08
**Relevant associated HRCT findings:**						
Pleural effusion	3	4.6%	7	10.8%	1.73	0.19
Pericardial effusion	2	3.1%	6	9.2%	2.13	0.14
Cavitation	1	1.5%	1	1.5%	0	1
Significant L.N enlargement	7	10.8%	5	7.7%	0.37	0.54

*P value < 0.05 is considered clinically significant

*Regarding other HRCT signs that were depicted in both COVID-19 and H1N1 patients, they are categorized as follows: (A) signs related to topographic picture of the diseases, (B) signs of lung parenchymal or alveolar involvement, (C) signs of airway involvement, and (D) signs related to pleuro-pericardial or nodal involvement.*

(A) Regarding the topographic HRCT findings

Prevalence rate of mixed peripheral and proximal lobular lung involvement was higher in H1N1 (35 patients, 53.8%) compared to COVID-19 (30 patients, 46.2%). On the other hand, prevalence rate of sub-pleural predilection was higher in COVID-19 (34 patients, 52.3%) compared to H1N1 (30 patients, 46.2%) (Table 2).

(B) Regarding the HRCT findings of parenchymal or alveolar involvement

**Fig. 3:**
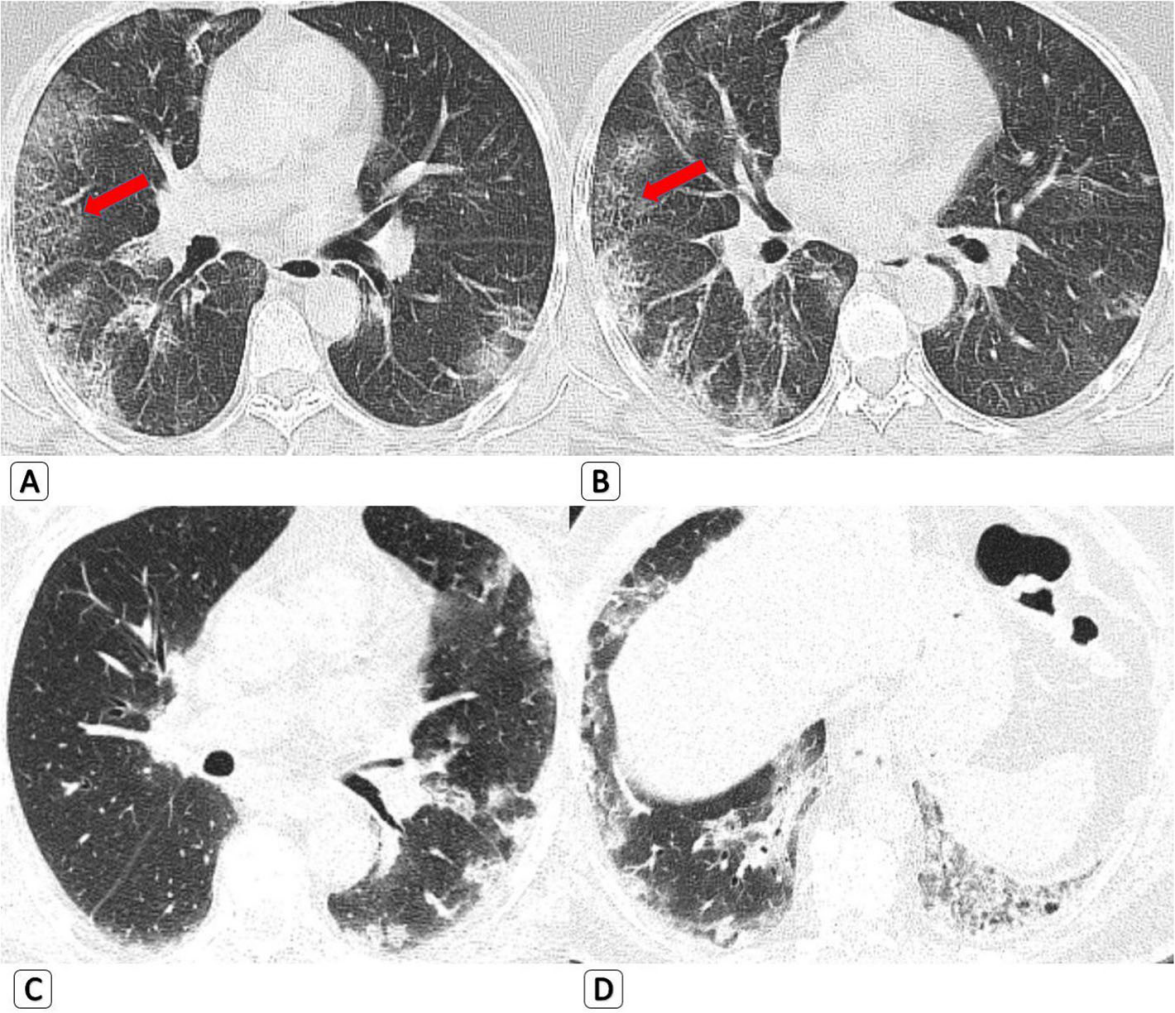
Pure parenchymal/alveolar pattern which cannot differentiate COVID19 from H1N1. **a**, **b** 44-year-old-male *COVID-19 patient* with axial HRCT chest lung window showing bilateral upper and lower lobar peripheral sub-pleural pure ground glass opacities. They shows smooth septal thickening on the right side “Crazy paving pattern” (red arrows). **c**, **d** 54-year-old male *H1N1 patient* with axial HRCT chest lung window showing bilateral upper and lower lobar mainly peripheral and sub-pleural dense ground glass patches

**Fig. 4:**
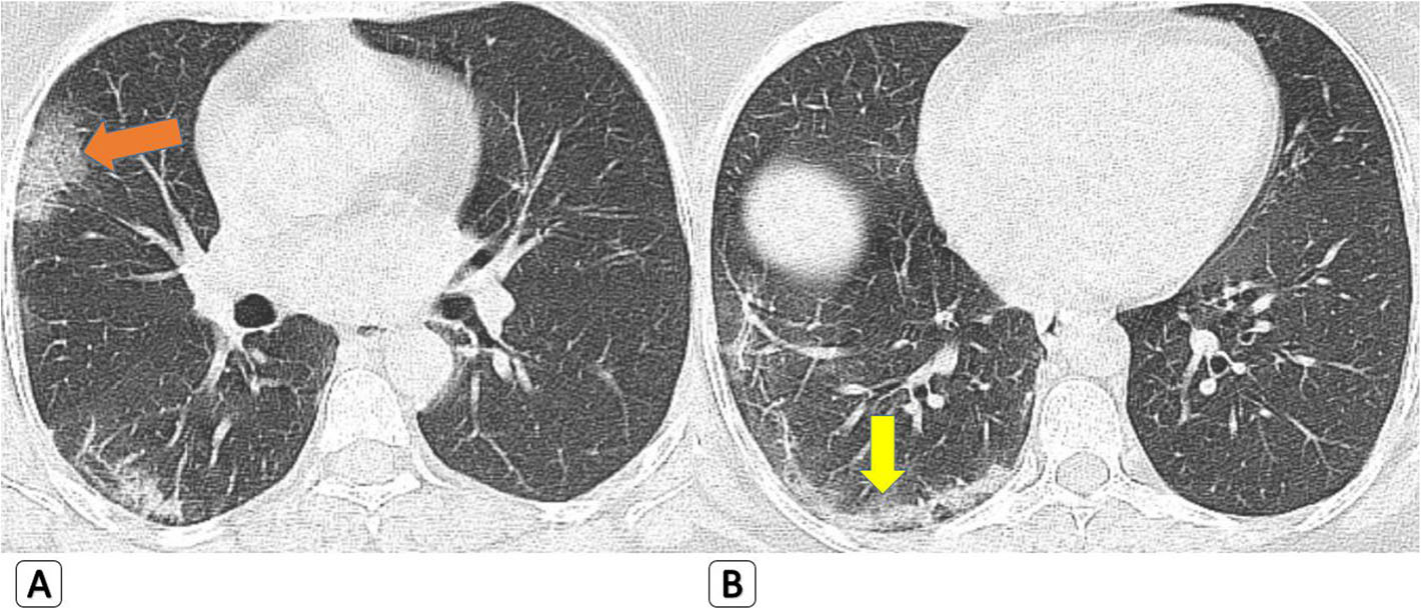
Mixed GGOs and “curvilinear organizing pattern of consolidation” seen with COVID-19. **a**, **b** 43-year-old female *COVID-19 patient* with axial HRCT chest lung window showing right sided upper lobar sub-pleural ground glass patchy showing “crazy paving pattern” (orange arrow) and lower lobar sub-pleural curvilinear pattern of organizing consolidation parallel to the pleural lining (yellow arrow)

**Fig. 5:**
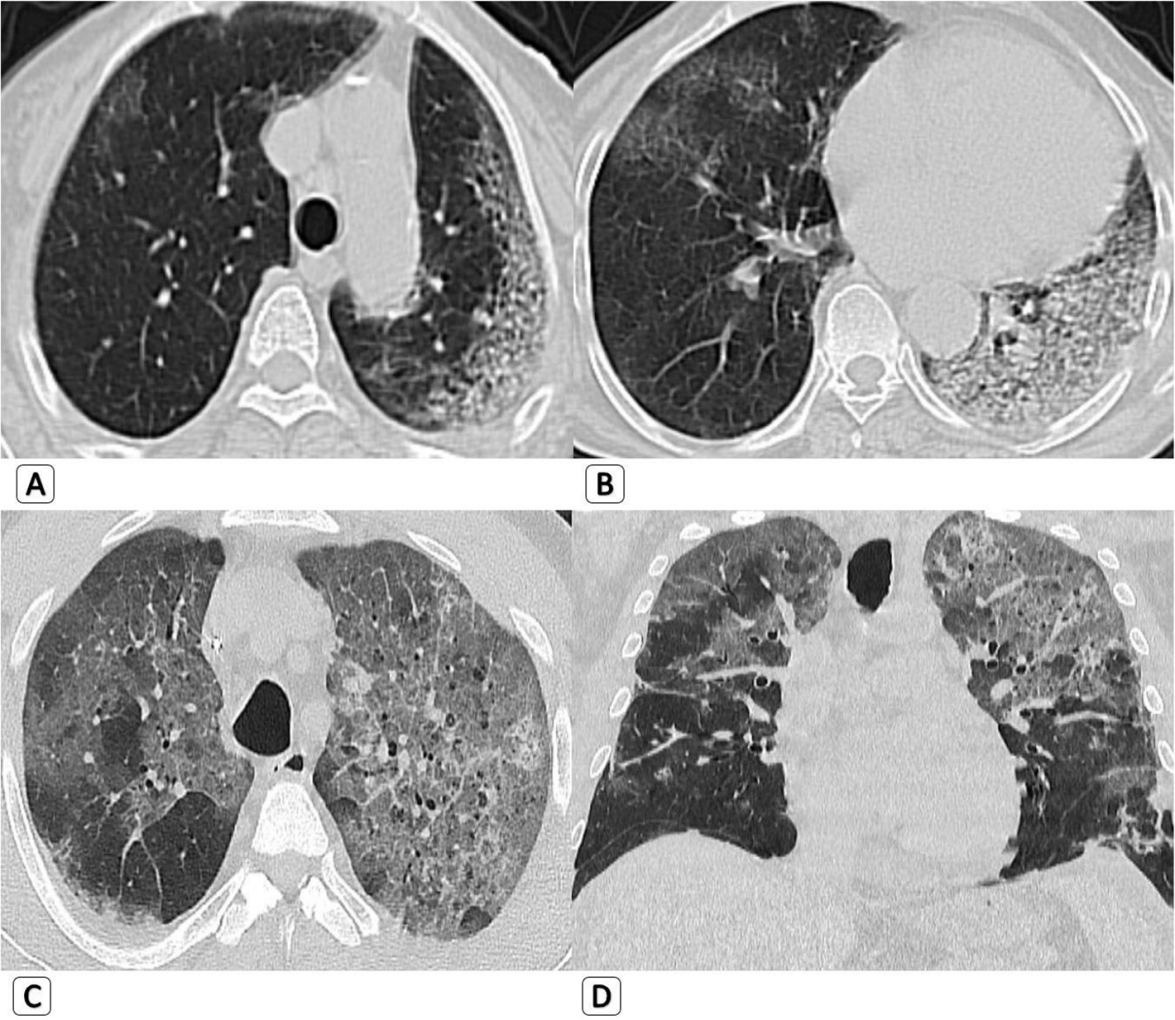
Severe cases complicated by diffuse alveolar damage (DAD), which cannot differentiate COVID19 from H1N1. **a**, **b** 52-year-old male *COVID-19 patient* with axial HRCT chest lung window showing bilateral ground glass opacities, more extensive and dense on the left side showing “crazy paving pattern” and mixed with mild air filled cystic changes denoting “early DAD.” **c**, **d** 58-year-old male *H1N1 patient* with axial and coronal HRCT chest lung window showing bilateral, predominantly upper lobar, ground glass opacity showing “crazy paving pattern” and mixed with air dilled cystic changes denoting “DAD”

GGOs were found in all COVID-19 patients and 90.8% of H1N1 patients. In mild cases, prevalence of pure ground glass nodules or patchy opacities (GGOs) was slightly higher in COVID-19 (18 patients, 27.7%) compared to H1N1 (10 patients, 15.4%) (Fig. 3). Prevalence of ground glass patches with peripheral organization “Atoll sign or reversed halo sign” as well as curvilinear bands was also higher in COVID-19 compared to H1N1 (Fig. 4). On the other hand, prevalence of mixed GGOs and consolidations was higher in H1N1 compared to COVID-19. Among severe or critical cases, prevalence of “crazy paving pattern” (with or without DAD or ARDS) was slightly higher in H1N1 compared to COVID-19 (Fig. 5). Cavitation was seen in one patient with H1N1 and COVID-19 (1.5%).

(C) Regarding the HRCT findings of airway involvement

Tree in bud nodules (sign of airway involvement) were seen in nine patients with H1N1 (13.8%) compared to only one patient with COVID-19 (1.5%). All of them had mild clinical presentation (Table 2).

(D) Regarding the other relevant HRCT findings

Pleural effusion and pericardial effusion, despite being uncommon CT finding and minimal in amount, were found more in H1N1 than COVID-19. Significant lymph node enlargement (short axis diameter exceed 1 cm) was noted in seven critical COVID-19 patients and five critical H1N1 patients (Table 2).

### Statistical analysis of CT performance

Only those HRCT findings which were corresponding to airway involvement and accompanied mild clinical presentation (namely tree in bud nodules, air trapping, bronchial wall thickening, traction bronchiectasis, and mucous plugging) showed significant *p* value (ranging from 0.008 to 0.04). On the other hand, other HRCT findings related to topographic features of the disease and other HRCT findings which were corresponding to parenchymal lung disease and accompanied both mild and severe clinical presentations showed non-significant *p* value > 0.05 (Table 2).

Among overall 65 patients proved with COVID-19, 20 patients were falsely diagnosed as H1N1. While among overall 65 patients proved with H1N1, 31 patients were falsely diagnosed as COVID-19. Accordingly and in overall, HRCT showed 50% sensitivity, 69.2% specificity, 61.5% positive predictive value (PPV), and 58.4% negative predictive value (NPV) for differentiation of H1N1 from COVID-19.

Among those nine patients proved with H1N1 having airway involvement (with or without parenchymal/alveolar HRCT findings), only one patient was falsely diagnosed as COVID-19. Meanwhile, the only one patient that had COVID-19 with mixed parenchymal and airway involvement was falsely diagnosed as H1N1. Accordingly, presence of HRCT findings of airway involvement in mild cases raise both HRCT sensitivity and PPV for differentiation of H1N1 from COVID-19 up to 88.9%.

**Fig. 6:**
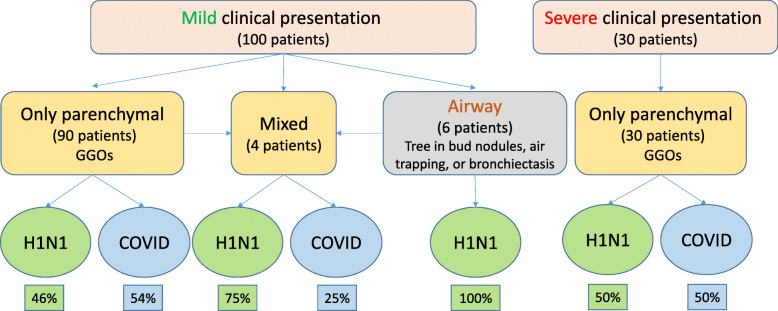
A schematic chart summarizing the results of the study: Presence of airway involvement in non-severe cases without or without parenchymal involvement favors the possibility of H1N1 over COVID-19. Otherwise, both possibilities would be equal if parenchymal involvement is noted alone in mild or severe cases

A schematic flow-chart is summarizing the results of the study among 100 mild patients and 30 severe patients (Fig. 6).

## Discussion

PCR tests for COVID-19 patients are not always positive and HRCT findings are not always pathognomonic [7, 8]. Hence, CT is recommended a routine examination during evaluation of COVID-19 [9]. As long as COVID-19 pandemic is continuous and flaring again during the current second wave, differential diagnosis of COVID-19 from other viral pneumonia will be a radiological challenge due to the overlap in clinical symptoms and laboratory signs. Accordingly, 65 patients with COVID-19 and other 65 patients with H1N1 were enrolled in this comparative radiological retrospective study in order to discover differentiating HRCT criteria that may ease this challenge.

Large and small airway involvement was strikingly missing in 98.5 % of COVID-19 patients who strictly presented by other HRCT findings related to pulmonary parenchymal or alveolar involvement. This is slightly different from Chen et al.’s [10] study which could not depict any sign of airway involvement in COVID-19 patients. On the other hand and approving Marchiori et al. [11], 13.8% of H1N1 patients showed specific HRCT findings consistent with large or small airway involvement whether isolated or mixed with parenchymal or alveolar pattern. Meanwhile, the rest of H1N1 patients (84.4%) presented by parenchymal involvement alone. Based on this finding and results of *p* value, the presence of airway involvement can highly suggest H1N1 viral pneumonia over COVID-19 after exclusion of past history of large or small airway disease such as asthma, COPD, or bronchiectasis.

This study matches Marchiori et al. [11] and Liu et al. [12], where bilateral mixed upper and lower lobar involvement was predominant in both COVID-19 and H1N1 patients. Peripheral sub-pleural predilection was predominant in COVID-19 patients while more diffuse involvement of both proximal and peripheral parts of the lungs was seen in H1N1 patients. Based on *p* value analysis, site of the pathology is non-differentiating parameter.

Regarding the lung parenchymal or alveolar pattern of the disease and keeping with Dawoud et al. [9], Liu et al. [12], and Schoen et al. [13], ground glass opacities (GGOs) were the most common HRCT findings among both COVID-19 and H1N1 patients, with or without consolidative changes. Similar to Hu et al. [14], “Atoll sign” was depicted in both COVID-19 and H1N1 patients, still with higher percentage in COVID19 (organizing pneumonia pattern). Based on *p* value analysis, HRCT findings of parenchymal or alveolar disease in mild patients are non-differentiating parameters. Among severe patients (mainly patients with DAD and ARDS), “crazy paving pattern” was also depicted in both COVID-19 and H1N1 patients, with slight higher percentage in H1N1. This is concordant with Pritt et al. [15] who reported that diffuse alveolar damage (DAD) was seen in severe cases among both COVID-19 and H1N1 patients, showing diffuse ground glass pattern and air filled cystic changes. Based on *p* value analysis, HRCT findings related to parenchymal or alveolar involvement in severe patients are non-differentiating parameters.

This study again go side by side to Marchiori et al. [11] regarding the HRCT findings of large or small airway involvement. Tree in bud nodules was the most common CT finding, seen in all H1N1 patients with airway involvement. Bronchial wall thickening and traction bronchiectasis with or without mucous plugging were noticed in half of involved H1N1 patients. Air trapping was also noted, with or without ground glass attenuation “head cheese sign”. Based on *p* value analysis, HRCT findings of airway disease in mild patients are good differentiating parameters.

Pleural effusion was noted in 10% of H1N1 patients, agreeing with Koo et al. [16] while only three patients with COVID-19 had pleural effusion sequel to cardiac and hepatic comorbidity. So presence or absence of pleural effusion is a non-differentiating parameter.

Pericardial effusion was seen in a small percentage of COVID-19 patients presenting with myocarditis, similar to findings of Inciardi et al. [17]. It was also found in a small percentage of H1N1 patients with ARDS, similar to findings of Al-Amoodi et al. [18]. Again presence or absence of pericardial effusion is a non-differentiating parameter.

This study disagreed with Bernheim et al. [19] that cavitation can exclude COVID-19 as found in one severe patient and hence agree with Sabri et al. [20]. On the other hand, it agreed with Khanna et al. [21] that cavitary changes could be found in with H1N1.

This study disagreed with Hu et al. [14] that lymph node enlargement can exclude both COVID-19 and H1N1, since significant enlargement was found among 33–50% of critical patients.

This study was limited by small number of patients proved with H1N1 at single center; hence, further large group future studies are encouraged.

## Conclusion

HRCT can help in differentiation between non-severe COVID-19 and H1N1 based on signs of airway involvement. Pulmonary parenchymal as well as pleural and pericardial involvement are not reliable differentiating CT parameters. Presence of cavitation or significant lymph node enlargement also are not reliable differentiating parameters. At severe stages, both COVID19 and H1N1 are considered as great mimics and non-differentiable on CT basis.

## Data Availability

The datasets used and/or analyzed during the current study are available from the corresponding author on reasonable request.

## Abbreviations

COVID 19: Coronavirus disease 2019
GGOs: Ground glass opacities
HRCT: High-resolution computed tomography
RT-PCR: Reverse transcription polymerase chain reaction
